# Association Between the Visceral Adiposity Index and Arterial Stiffness: Results of the EVasCu Study and a Meta-Analysis Including EVasCu Data and Prior Studies

**DOI:** 10.3390/metabo16010020

**Published:** 2025-12-24

**Authors:** Elena Rescalvo-Fernández, Iván Cavero-Redondo, María Medrano, Irene Martínez-García, Carla Geovanna Lever-Megina, Marta Fenoll-Morante, Alicia Saz-Lara

**Affiliations:** 1 Hospital Universitario La Paz, 28046 Madrid, Spain; elena.rescalvo.fernandez@gmail.com; 2 CarVasCare Research Group, Faculty of Nursing, University of Castilla-La Mancha, 16001 Cuenca, Spain; irene.mgarcia@uclm.es (I.M.-G.); carlageovanna.lever@uclm.es (C.G.L.-M.); marta.fenoll@alu.uclm.es (M.F.-M.); alicia.delsaz@uclm.es (A.S.-L.); 3 Institute for Innovation & Sustainable Food Chain Development, Department of Health Sciences, Public University of Navarra, 31009 Pamplona, Spain; maria.medrano.echeverria@gmail.com; 4 CIBER de Fisiopatología de la Obesidad y Nutrición (CIBEROBN), Instituto de Salud Carlos III, 28029 Madrid, Spain

**Keywords:** visceral adipose index, arterial stiffness, pulse wave velocity, cardiovascular risk, adult population

## Abstract

**Objectives**: This study aimed to examine the association between the visceral adiposity index and arterial stiffness in healthy adults via original data from the EVasCu study and to contextualize these findings through a meta-analysis of previously published studies in the general population. **Methods**: A cross-sectional analysis was conducted in 389 healthy adults from the EVasCu study. The visceral adiposity index was calculated on the basis of waist circumference, body mass index, triglycerides, and high-density lipoprotein cholesterol, integrating the anthropometric and metabolic components of visceral adiposity. Arterial stiffness was assessed by the aortic pulse wave velocity. These original findings were complemented by a meta-analysis, including EVasCu data and data from prior studies, to obtain pooled correlation coefficients and 95% confidence intervals (CIs) for the association between visceral adiposity and arterial stiffness. **Results**: In the EVasCu study, the visceral adiposity index showed a statistically significant moderate correlation with the aortic pulse wave velocity (r = 0.281, *p* < 0.001). In the meta-analysis, the pooled correlation coefficient was 0.34 (95% CI: 0.27, 0.42), supporting a consistent association between the visceral adiposity index and both central and peripheral arterial stiffness across diverse populations. **Conclusions**: These findings indicate a positive association between the visceral adiposity index and arterial stiffness in both healthy individuals and populations with cardiometabolic conditions. However, given the predominantly cross-sectional nature of the evidence and the heterogeneity among the included studies, the results should be interpreted with caution. Further longitudinal, multivariable, and mechanistic studies are needed to clarify the clinical relevance of the visceral adiposity index beyond correlation and to determine its potential role as a complementary marker in cardiovascular risk assessment.

## 1. Introduction

Cardiovascular disease (CVD) remains the leading cause of mortality worldwide, accounting for approximately 17.9 million deaths annually [1]. Given this burden, early identification of vascular alterations prior to the development of overt clinical disease is essential for improving preventive strategies. In this context, arterial stiffness denotes alterations in the mechanical properties of large arteries, characterized by a diminished capacity to buffer pulsatile pressure and maintain normal hemodynamic function, and has emerged as a robust biomarker of subclinical vascular damage and a strong predictor of future cardiovascular events [2,3,4].

Among the available noninvasive assessment methods, pulse wave velocity (PWV) is widely accepted as the most informative noninvasive indicator of arterial wall stiffness because it directly quantifies the speed at which pressure waves propagate along the arterial tree [5,6,7]. Increasing arterial stiffness reflects cumulative structural and functional vascular injury and is recognized as an early manifestation of early vascular ageing (EVA), preceding the clinical onset of major cardiometabolic disorders. Consequently, PWV consistently predicts the development of hypertension, diabetes, atherosclerosis, and cardiovascular morbidity and mortality, even in young and apparently healthy individuals, highlighting its value for detecting very early vascular impairment [5,6,7].

Obesity, particularly excess visceral adiposity, plays a key role in cardiometabolic dysfunction and is strongly associated with endothelial impairment, arterial remodelling, and atherosclerotic burden. Traditional anthropometric indices, such as body mass index (BMI), waist circumference (WC), and the waist-to-hip ratio, are widely used in clinical practice; however, these indices do not adequately distinguish between subcutaneous and visceral fat depots and therefore provide limited information on adipose tissue dysfunction [8]. To address these limitations, Amato et al. developed the visceral adipose index (VAI), a sex-specific indicator that integrates anthropometric measures (WC and BMI) and metabolic parameters (triglycerides [TGs] and high-density lipoprotein cholesterol [HDL-c]). Compared with BMI or WC alone, the VAI has demonstrated stronger associations with insulin resistance, metabolic syndrome, and cardiometabolic risk, suggesting improved sensitivity for detecting dysfunctional visceral adiposity [9]. In addition to the VAI, other adiposity-related indices have been explored in relation to vascular health. One example is the body shape index (ABSI), which captures abdominal body shape using waist circumference adjusted for height and BMI. ABSI has been shown to be associated with arterial stiffness in previous research [10], although it does not incorporate metabolic information such as triglycerides or HDL-c. This distinction is relevant because vascular ageing is strongly influenced by metabolic dysfunction, supporting the rationale for examining the VAI as a complementary and potentially more physiologically informative marker.

Although increasing evidence suggests that higher VAI values are associated with adverse metabolic and cardiovascular profiles, research specifically examining the relationship between the VAI and arterial stiffness remains scarce. While several studies have reported associations between traditional anthropometric markers and arterial stiffness [8,11,12,13,14,15], findings regarding the VAI are limited and heterogeneous, and most studies have focused on populations with established cardiometabolic conditions, limiting inferences about early vascular alterations. Importantly, evidence in healthy individuals is extremely scarce, which restricts our understanding of whether visceral adiposity dysfunction contributes to early vascular damage before the onset of clinical disease. Moreover, to date, no systematic review or meta-analysis has comprehensively synthesized the available evidence on the association between the VAI and arterial stiffness.

In this context, PWV represents an optimal vascular outcome for exploring early links between visceral adiposity and vascular aging because of its strong physiological relevance and prognostic value. Even modest correlations between the VAI and PWV in young adults may reflect early pathological changes, underscoring the need to investigate this association in healthy populations.

Therefore, the aims of this study were as follows:

1. To analyse the association between the VAI and arterial stiffness, measured by the aortic PWV (a-PWV), in healthy subjects from the EVasCu study;
2. To contextualize these findings by conducting a meta-analysis including EVasCu data and previous studies evaluating the association between the VAI and arterial stiffness (both central and peripheral) in healthy and clinical populations, formally assessing and exploring heterogeneity across studies.

## 2. Materials and Methods

### 2.1. EVasCu Study

#### 2.1.1. Design, Participants, and Sample Size

The EVasCu study is a cross-sectional study conducted in healthy adults living in the province of Cuenca (Spain) with the aim of characterizing early markers of vascular ageing in apparently healthy individuals. The participants were adults without previously diagnosed chronic diseases. Among the 390 individuals initially recruited, 389 were included in the present analysis, as one participant lacked complete biochemical data.

Sample size estimation was performed a priori via Epidat software, version 4.2. The calculation indicated that a minimum of 355 participants would be required to detect an estimated effect size of 1, with an alpha risk of 0.05 and an absolute precision of 0.04 for the EVA index [16]. On the basis of these assumptions, the final sample of 390 participants was considered adequate to detect statistically significant associations.

The study was conducted and reported in accordance with the Strengthening the Reporting of Observational Studies in Epidemiology (STROBE) guidelines [17].

#### 2.1.2. Ethical Considerations

The study protocol was approved by the Clinical Research Ethics Committee of the Cuenca Health Area (REG:2022/PI2022). Written informed consent was obtained from all participants prior to enrolment. The study was conducted in accordance with the principles of the Declaration of Helsinki.

#### 2.1.3. Variables

Arterial stiffness was assessed via a validated oscillometric device (Mobil-O-Graph^®^, IEM GmbH, Stolberg, Germany), which derives the aortic pulse wave velocity (a-PWV) from the contour of the brachial pressure waveform. This device has been previously validated against invasive and noninvasive reference methods and has demonstrated acceptable accuracy and reproducibility for assessing central PWV [18]. Measurements were performed in a standardized setting: a quiet room, after a 5 min seated rest, and appropriately sized cuffs were used. Two consecutive recordings separated by 5 min were obtained for each participant, and their mean value was used in the analyses.

Visceral adiposity was estimated via the VAI, which incorporates anthropometric parameters such as WC and BMI, in addition to biochemical markers such as TG and HDL-c. The following sex-specific formulas were applied:

$$VAI_{\mathrm{Men}}=\left(\frac{WC}{39.68+(1.88\times BMI)}\right)\times \left(\frac{TG}{1.03}\right)\times \left(\frac{1.31}{HDL}\right)$$

$$VAI_{\mathrm{Women}}=\left(\frac{WC}{36.58+(1.89\times BMI)}\right)\times \left(\frac{TG}{0.81}\right)\times \left(\frac{1.52}{HDL}\right)$$

For anthropometric assessments, WC was measured twice at the end of normal expiration at the midpoint between the lowest rib and the iliac crest via flexible tape, and the average was recorded. Height was measured barefoot with a wall stadiometer (Seca^®^ 222, Hamburg, Germany), and weight was obtained with a calibrated scale to compute BMI (kg/m^2^).

TG and HDL-c concentrations were determined from fasting venous blood samples via high-performance liquid chromatography.

Although the PWV and VAI were defined a priori as the primary variables of interest, additional cardiometabolic variables, such as systolic and diastolic blood pressure, lipid profile components (total cholesterol, LDL-c), glucose levels, and smoking status, were also collected to characterize the sample, even if not included in the primary correlation analysis.

#### 2.1.4. Data Analysis of the EVasCu Study

The distribution of continuous variables was examined through probability plots and the Kolmogorov–Smirnov test. Linearity assumptions were evaluated prior to correlation analyses. Descriptive statistics are presented as the means and standard deviations (SDs) or percentages (%), as appropriate. The association between a-PWV and the VAI was explored via Pearson’s correlation coefficient.

Analyses were conducted via IBM SPSS 28 (SPSS Inc., Chicago, IL, USA), with statistical significance set at *p* < 0.05.

### 2.2. Systematic Review and Meta-Analysis

This systematic review and meta-analysis was conducted following the Meta-Analysis of Observational Studies in Epidemiology (MOOSE) statement [19] and followed the guidelines described in the Cochrane Collaboration Handbook [20]. The study protocol was registered in PROSPERO (CRD42023473126).

#### 2.2.1. Search Strategy

A comprehensive search was performed in Scopus, PubMed, and Web of Science from inception to October 2023. Both Medical Subject Headings (MeSH) and free-text terms were combined via Boolean operators (AND/OR) following the PICO strategy (population, intervention/exposure, comparison, outcome): “adults”, “adult population”, “adult subjects”, “visceral adiposity index”, “arterial stiffness”, “pulse wave velocity”, “new visceral adipose index”, “brachial-ankle pulse wave velocity”, “aortic pulse wave velocity” and “carotid-femoral pulse wave velocity”. No language or publication date restrictions were applied. Database-specific adaptations of search terms were used. A final search was conducted prior to analysis to identify newly published studies. Supplementary Table S1 displays the standardized search strategy used for the databases.

#### 2.2.2. Inclusion and Exclusion Criteria

Studies were included if they met the following criteria: (i) adult population (≥18 years); (ii) exposure: VAI; and (iii) outcome: central arterial stiffness (measured by a-PWV or carotid–femoral PWV [cf-PWV]) and peripheral arterial stiffness (measured by brachial-ankle PWV [ba-PWV]). Studies with mixed populations were included if cross-sectional associations were reported. Reviews, editorials, and studies without cross-sectional data were excluded.

#### 2.2.3. Data Extraction and Quality Assessment

Data extraction was independently performed by two reviewers (ER-F and IC-R) who were blinded to the study hypotheses. Study quality was assessed via the National Institutes of Health (NIH) Quality Assessment Tool for Observational Cohorts and Cross-Sectional Studies [21], with particular attention given to confounding control, exposure and outcome measurement, and statistical methods. Discrepancies were resolved by consensus or consultation with a third reviewer (AS-L).

#### 2.2.4. Statistical Analysis

The DerSimonian and Laird random-effects method [22] was used to calculate pooled estimates of correlation coefficients (r) along with their respective 95% confidence intervals (CIs) to evaluate the relationship between arterial stiffness and the VAI. Heterogeneity was evaluated via the I2 statistic, which ranges from 0 to 100%. Depending on the I2 values, heterogeneity was classified as unimportant (0–30%), moderate (30–60%), substantial (60–75%), or considerable (75–100%) [23]. The corresponding *p* values were also considered in the analysis.

Sensitivity analysis was conducted to assess the robustness of the summary estimates through systematic reanalysis by excluding one study at a time. Subgroup analyses were performed on the basis of the type of arterial stiffness (central [a-PWV, cf-PWV] or peripheral [ba-PWV]). Exploratory random effects meta-regression analyses were used to examine whether variables such as the mean age, percentage of women, WC, BMI, TG, and HDL-c modified the associations between central and peripheral arterial stiffness and the VAI. Additionally, publication bias was evaluated via Egger’s regression asymmetry test [24], with a significance level of <0.10 indicating the presence of publication bias.

Statistical analyses were performed via STATA SE software, version 15 (StataCorp, College Station, TX, USA).

## 3. Results

### 3.1. EVasCu Study Results

#### 3.1.1. Characteristics of the Participants

Among the 390 participants enrolled in the EVasCu study, 389 were included in the present analysis (of whom 143 were men and 246 were women), as one participant was excluded because of missing biochemical marker data. The mean age of the participants was 42.03 ± 13.2 years. The baseline characteristics of the participants are shown in Table 1.

#### 3.1.2. Associations Between Arterial Stiffness and the Visceral Adipose Index

**Figure 1:**
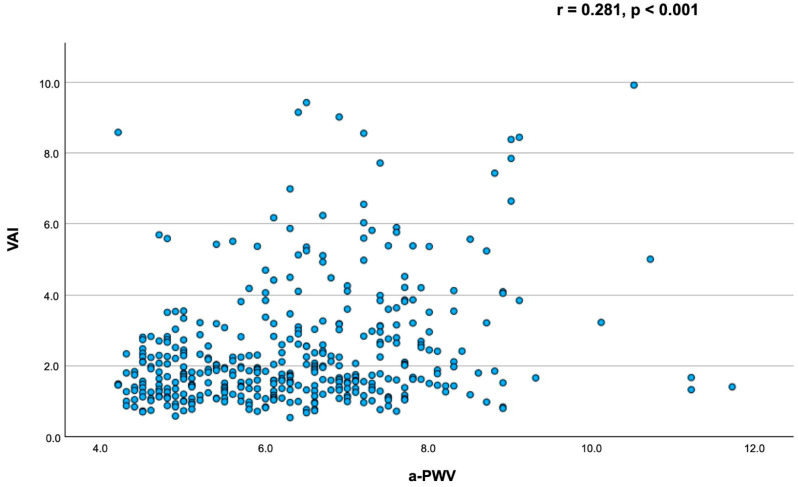
Associations between the aortic pulse wave velocity and the visceral adiposity index.

The correlation coefficient for the association between a-PWV and the VAI was 0.281 (*p* < 0.001) (Figure 1).

### 3.2. Systematic Review and Meta-Analysis

#### 3.2.1. Characteristics of the Included Studies

**Figure 2:**
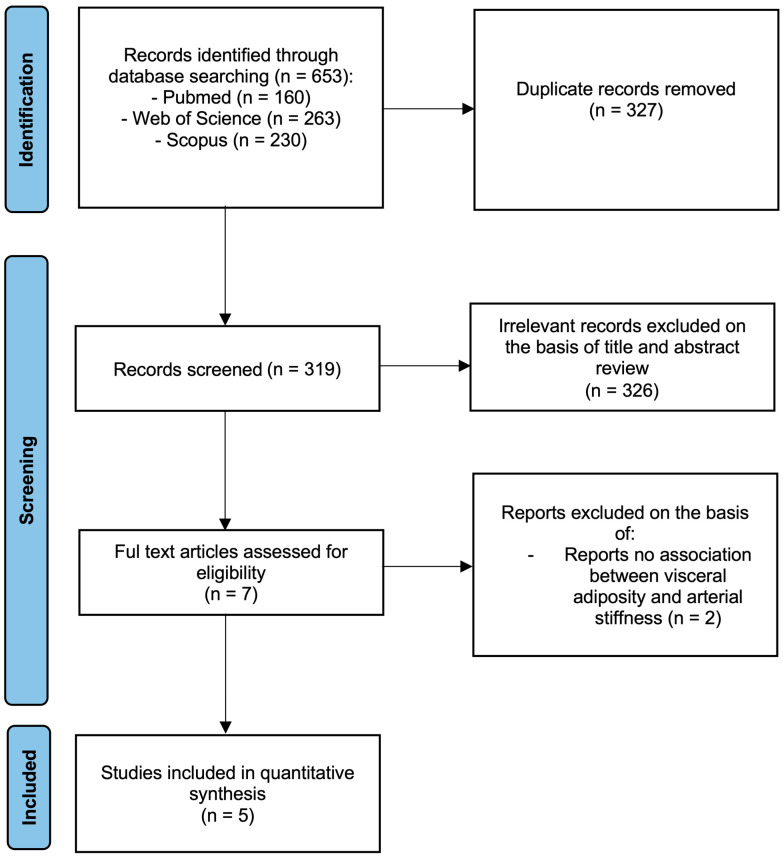
Flowchart.

In total, 5 studies were included in the systematic review and meta-analysis, along with the EVasCu study (6 studies in total). Two potentially relevant studies were excluded because they did not provide extractable cross-sectional correlation coefficients or sufficient statistical information to compute a correlation coefficient (Figure 2). Among the included studies, 5 were cross-sectional [11,12,13,14,15], and one was a retrospective longitudinal study [8]. Of the six included studies, two were conducted in China [11,12], one in Korea [8], one in Japan [11], one in Iran [14], and one in Spain [15]. The studies were published between 2019 and 2023 and included a total of 75,031 subjects aged 38.8 to 67.4 years. The studies were conducted in the general population and included healthy participants and those with different comorbidities, such as DM, HT, and dyslipidaemia, and risk factors, such as smoking.

Finally, different methods were used to measure arterial stiffness: two for central arterial stiffness (one study for cf-PWV [14], one study for a-PWV [15], and four for peripheral arterial stiffness [ba-PWV]) [8,11,12,13]. Table 2 shows the characteristics of the included studies.

#### 3.2.2. Quality Assessment

The overall risk of bias across studies investigating the correlation between the VAI and central arterial stiffness was good in 100% of the included studies. The overall risk of bias for studies examining the association between the VAI and peripheral arterial stiffness was good in 75% and fair in 25% of the included studies (Supplementary Table S2). For all exposures, we were able to identify three main reasons for a reasonable risk of bias: (i) no justification of sample size was reported; (ii) most studies were cross-sectional, the items referring to follow-up could not be assessed; and finally, (iii) most studies did not report whether the investigators were blinded to the participants’ exposure status.

#### 3.2.3. Association Between Arterial Stiffness and the Visceral Adipose Index

**Figure 3:**
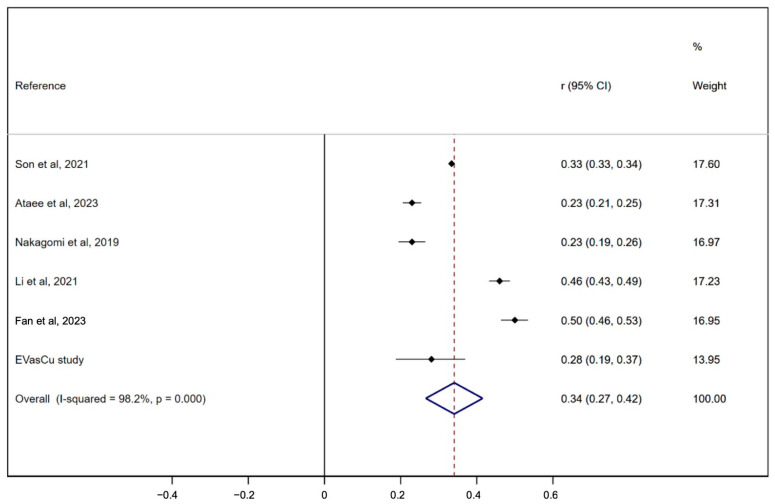
Forest plot including the association between the visceral adiposity index and arterial stiffness [8,11,12,13,14,15]. The dots reflect the estimated mean value of each study, the lines the 95% confidence intervals, and the diamond the pooled value of all included studies.

Figure 3 shows the combined correlation coefficients between central and peripheral arterial stiffness and the VAI. The pooled estimate for the correlation coefficient was 0.34 (95% CI: 0.27, 0.42) for arterial stiffness and the VAI, indicating considerable heterogeneity (I2: 98.2%).

#### 3.2.4. Sensitivity Analysis

Pooled estimates of the correlation coefficient for the association between central and peripheral arterial stiffness and the VAI remained stable (in magnitude or direction) when individual study data were removed one by one from the analysis.

#### 3.2.5. Subgroup Analysis, META-Regression Models and Publication Bias

Subgroup analyses according to arterial stiffness type revealed that the pooled correlation coefficient estimate was 0.24 (95% CI: 0.20, 0.27) for central arterial stiffness and the VAI, with no important heterogeneity (I2: 10.4%), and 0.38 (95% CI: 0.29, 0.48) for peripheral arterial stiffness and the VAI, with considerable heterogeneity (I2 = 98.4%) (Supplementary Figure S1). Random effects meta-regression models indicated that age significantly influenced the combined correlation coefficient estimate for the relationship between arterial stiffness and the VAI (*p* = 0.001). (Supplementary Table S3). Additionally, Egger’s test revealed no evidence of publication bias regarding the association between arterial stiffness and the VAI (*p* = 0.883).

## 4. Discussion

The present study assessed the association between the VAI and arterial stiffness in healthy adults from the EVasCu study and contextualized these findings through a meta-analysis including EVasCu data and prior studies. In the EVasCu study, we observed a statistically significant positive correlation between the VAI and a-PWV, indicating that visceral adipose dysfunction is associated with early vascular alterations even in asymptomatic individuals. The meta-analysis further confirmed this association for both central and peripheral arterial stiffness, supporting the consistency of the relationship across populations and measurement approaches.

### 4.1. Interpretation and Clinical Relevance of the Association

Importantly, the magnitude of the observed associations should be interpreted with caution. Both in the EVasCu study and in the pooled meta-analytic estimates, the correlations were small to moderate in strength. While modest, such associations are clinically meaningful in the context of early vascular ageing, where subtle changes in arterial stiffness may precede overt cardiovascular disease by several years. These findings suggest that the VAI may capture early metabolic–vascular interactions that are not fully reflected by traditional anthropometric measures.

### 4.2. Contextualization Within Previous Literature

Previous studies have consistently shown that traditional anthropometric markers, BMI, WC, and the waist-to-hip ratio are associated with cardiovascular risk [25,26]. However, their limited ability to differentiate between visceral fat and subcutaneous fat reduces their pathophysiological specificity. In contrast, the VAI integrates both anthropometric and metabolic components (TG and HDL-c), providing a more refined estimate of visceral adipose dysfunction [8,9]. Several prior studies have reported positive associations between the VAI and arterial stiffness assessed by ba-PWV or cf-PWV [8,11,12,13,14,15], with some suggesting stronger associations for the VAI than other indices of abdominal obesity [14]. Our findings are in agreement with this literature and extend it by demonstrating similar associations in a well-characterized cohort of healthy adults. Our findings also contribute to broader discussions of anthropometric indices and vascular aging. While previous research using ABSI has reported associations with arterial stiffness [10], ABSI primarily reflects body geometry and does not account for lipid-related dysfunction. Considering that visceral adipose dysfunction is a key driver of inflammation, endothelial impairment, and arterial remodelling, the VAI may offer additional mechanistic relevance compared with morphology-based indices.

### 4.3. Relevance of the PWV and VAI for Early Vascular Aging

Aortic PWV was selected as the primary vascular outcome because of its established role as a gold-standard marker of arterial stiffness and cumulative vascular damage, with strong prognostic value for cardiovascular events. Similarly, the VAI was chosen because visceral adiposity is a key driver of inflammation, endothelial dysfunction, and metabolic dysregulation, processes that contribute directly to vascular stiffening. Together, PWV and VAI offer a physiologically coherent framework for exploring early vascular alterations in populations without overt disease, where prevention efforts may be most effective.

### 4.4. Potential Biological Mechanisms

Several interrelated mechanisms may underlie the association between visceral adiposity and arterial stiffness. Visceral fat is metabolically active and promotes a proinflammatory milieu characterized by increased secretion of cytokines and adipokines, leading to oxidative stress, insulin resistance, and endothelial dysfunction [27,28,29,30,31]. These processes may reduce nitric oxide bioavailability, activate the renin–angiotensin–aldosterone system, and promote glycation of extracellular matrix proteins, ultimately resulting in vascular wall hypertrophy and fibrosis [29,32,33,34,35]. While our findings are consistent with these pathways, the cross-sectional nature of the evidence precludes mechanistic inference, underscoring the need for targeted experimental and longitudinal studies.

### 4.5. Effect Modifiers and Population Differences

Age emerged as a potential modifier of the VAI–arterial stiffness association in our meta-analysis, which is consistent with previous reports indicating stronger associations in older adults [8,13,14]. Sex-related differences have been observed in prior studies [11,15,36], possibly reflecting differences in fat distribution and hormonal status, although such differences were not detected in the EVasCu study. Ethnic variability has also been suggested, with stronger associations reported in Asian populations [14,37,38], likely due to differences in visceral fat accumulation. However, ethnic differences were not directly assessed in the present analyses and are discussed in light of the literature rather than the original subgroup findings.

### 4.6. Analytical Scope and Study Focus

The analysis of this study of the EVasCu study was designed to test a predefined hypothesis focusing on the association between the VAI and PWV. Although additional cardiometabolic variables were collected, extending correlation analyses to all measured parameters was beyond the scope of the primary research question and could have diluted interpretability. Future multivariable and integrative analyses may provide further insights into the relative contribution of the VAI within broader cardiometabolic risk profiles.

### 4.7. Limitations and Future Directions

Several limitations should be acknowledged. First, the cross-sectional design of both the EVasCu study and most of the studies included in the meta-analysis precludes establishing causality or evaluating the predictive capacity of the VAI for future vascular changes. Second, although the sample size of the EVasCu study was adequate, larger studies would improve precision and allow for more robust subgroup analyses. Third, residual confounding cannot be excluded, particularly given that the meta-analysis relied on unadjusted correlation coefficients derived from heterogeneous observational studies. Fourth, the participants were drawn from a specific Spanish region, which may limit generalizability to populations with different sociodemographic or ethnic characteristics. Fifth, in the meta-analysis, substantial heterogeneity was observed in studies using carotid–femoral PWV, likely reflecting variability in population characteristics and measurement protocols. Finally, the limited number of studies assessing central arterial stiffness restricts the depth of evidence at this vascular level.

Future research should prioritize longitudinal cohort designs, standardized PWV assessment protocols, and interventional studies to clarify temporality, causality, and the potential clinical utility of the VAI as a complementary marker of early vascular ageing.

## 5. Conclusions

On the basis of the findings of the EVasCu study and the accompanying meta-analysis, our results suggest a positive association between the VAI and arterial stiffness in both healthy individuals and populations with cardiometabolic comorbidities. These findings should be interpreted as preliminary, as the predominantly cross-sectional nature of the available evidence precludes causal inference or confirmation of predictive capacity. While the VAI may represent a useful complementary marker for identifying individuals with early vascular aging, its clinical application should be considered cautiously. Further longitudinal, multivariable, and mechanistic studies in diverse populations are needed to clarify temporal relationships and underlying biological pathways and to determine whether the VAI provides incremental value beyond established anthropometric and biochemical markers in cardiovascular prevention strategies.

## Figures and Tables

**Table 1:** Characteristics of the participants included in the EVasCu study.

	Total (n = 389)
**Age (years)**	42.03 ± 13.2
**Sex, n (%)**	
Male	143 (36.8)
Female	246 (63.2)
**Anthropometric measurements**	
Waist circumference (cm)	82.6 ± 12.8
Body mass index (kg/m^2^)	24.8 ± 4.2
Visceral adiposity index (VAI)	2.4 ± 1.7
**Biochemical measurements**	
HDL- Cholesterol (mg/dL)	61.6 ± 13.7
Triglycerides (mg/dL)	86.1 ± 43.3
**Arterial stiffness**	
Aortic pulse wave velocity (m/s)	6.3 ± 1.4

**Table 2:** Characteristics of the studies included in the systematic review and meta-analysis.

Reference	Country	Study Design	Sample Size (n. % Female)	Mean Age (Years)	Type of Population	Baseline VAI Levels	PWV Measurement Device	Type of PWV	Baseline PWV Levels (m/s)
Nakagomi et al. 2019 [11]	Japan	Cross-sectional	2818 (1098. 38.96)	**M**: 38.8 ± 10.1 **F**: 39.1 ± 9.4	DM	**M**: 51.8 ± 52.5 **F**: 39.4 ± 32.3	Omron Colin.	ba-PWV	**M**: 12.25 ± 1.83 **F**: 10.94 ± 1.68
Son et al. 2021 [8]	Korea	Cross-sectional data from longitudinal study	60,938 (16,682. 27.38)	**M**: T1: 46.4 ± 8.9 T2: 51.7 ± 8.6 T3: 55.9 ± 9.3 **F**: T1: 44.5 ± 9 T2: 53.7 ± 8.0 T3: 59.7 ± 8.4	Healthy HT DM DL Smokers Athletes	**M**: T1 (≤ 0.27) 14.765 T2 (0.28–0.75) 14.771 T3 (>0.75) 14.765 **F**: T1 (≤0.02) 5560 T2 (0.03–0.18) 5563 T3 (>0.18) 5559	Plethysmographic.	ba-PWV	**M**: T 1 (≤0.27) 6.10 ±0.041 T 2 (0.28–0.75) 10.87 ± 0.074 T 3 (>0.75) 16.15 ± 0.109 **F**: T 1 (≤0.02) 1.51 ± 0.027 T 2 (0.03–0.18) 3.80 ± 0.068 T 3 (>0.18) 4.88 ± 0.088
Li et al. 2021 [12]	China	Cross-sectional	3258 (1614. 49.5)	Q1: 66.9 ± 9.0 Q2: 66.4 ± 9.2 Q3: 65.3 ± 8.8 Q4: 63.5 ± 9.0	HT	Q1: 0.6 ± 0.2 Q2: 1.1± 0.2 Q3: 1.8 ± 0.3 Q4: 4.4 ± 3.5	Omron Colin BP-203RPE III.	ba-PWV	Q1: 17.9 ± 3.7 Q2: 18.1 ± 4.0 Q3: 18.5 ± 3.98 Q4: 18.5 ± 4.0
Fan et al. 2023 [13]	China	Cross-sectional	1707 (566. 33.2)	67.4 ± 6	HT DM DL Healthy Smokers	T1 (0.22–0.99) 561 T2 (1.00–1.74) 576 T3 (1.75–5.95) 570 Total 1.31 (0.85–2.04)	Omron Colin BP-202 RPE III.	ba-PWV	**baPWV < 14 m/s** T1 (0.22–0.99) 1.00 T2 (1.00–1.74) 0.58 T3 (1.75–5.95) 0.70 **baPWV ≥ 14 m/s** T1 (0.22–0.99) 4.64 T2 (1.00–1.74) 5.16 T3 (1.75–5.95) 4.99
Ataee et al. 2023 [14]	Iran	Cross-sectional	5921 (3109. 52.5)	**M:** UW: 46.3 ± 13.6 N: 46.9 ± 11.2 OW: 46.9 ± 10.5 O: 47.6 ± 9.9 Total: 47.3 ± 10.67 **F:** UW: 40.5 ± 9.6 N: 41.1 ± 8.3 OW: 44.6 ± 8.9 O: 46.7 ± 9.7 Total: 43.66 ± 9.14	HT DM DL Healthy Smokers	**M**: UW: 2.04 ± 1.01 N: 3.38 ± 2.82 OW: 4.51 ± 3.32 O: 4.66 ± 3.04 **F**: UW: 2.20 ± 1.3 N: 3.1 ± 2.2 OW: 3.97 ± 2.5 O: 4.83 ± 3.1	SphygmoCor XCEL	cf-PWV	**M**: UW: 6.05 ± 0.8 N: 7.2 ±1.47 OW: 7.70 ± 1.58 O: 8.16 ± 1.72 Total: 7.5 ± 1.6 **F**: UW: 5.93 ± 1.04 N: 6.24 ± 1.22 OW: 6.78 ± 1.45 O: 7.40 ± 1.73 Total: 6.6 ± 1.5
Estudio EVasCu et al. 2023 [15]	Spain	Cross-sectional	389(246. 63.2)	42.03 ± 13.2	Healthy	2.4 ± 1.7	Mobil-O-Graph	a-PWV	6.3 ± 1.4

Legend: A-PWV: aortic pulse wave velocity; Ba-PWV: brachial–ankle pulse wave velocity; Cf-PWV: carotid–femoral pulse wave velocity; DL: dyslipidaemia; DM: diabetes mellitus; F: Female; HT: hypertension; M: male; N: normal; O: obesity; OW: overweight; PWV: pulse wave velocity; Q: quartile; T: tertile; UW: underweight; VAI: visceral adipose index.

## Data Availability

The original contributions presented in the study are included in the article, and further inquiries can be directed to the corresponding author.

## Supplementary Materials

The following supporting information can be downloaded at https://www.mdpi.com/article/10.3390/metabo16010020/s1. Supplementary Table S1. Search strategy; Supplementary Table S2. Quality assessment with the tool for observational cohort and cross-sectional studies of the National Heart, Lung, and Blood Institute for the association between arterial stiffness and the visceral adiposity index; Supplementary Table S3: Meta-regression models according to mean age, percentage of females, waist circumference, body mass index, HDL-cholesterol, and triglycerides; Supplementary Figure S1. Forest plot including the associations between the visceral adiposity index and central and peripheral arterial stiffness.

## Abbreviations

The following abbreviations are used in this manuscript:

CVD	Cardiovascular disease
HT	Hypertension
DM	Diabetes mellitus
BMI	Body mass index
WC	Waist circumference
VAI	Visceral adipose index
HDL-c	High-density lipoprotein cholesterol
TG	Triglycerides
SDs	Standard deviation
CIs	Confidence intervals
PICO	Population, intervention, comparator, outcome
MOOSE	Meta-analysis of observational studies in epidemiology
a-PWV	Aortic pulse wave velocity
ba-PWV	Brachial–ankle pulse wave velocity
cf-PWV	Carotid–femoral pulse wave velocity
PVW	Pulse wave velocity
