# Supplementary material for: Association Between the Visceral Adiposity Index and Arterial Stiffness: Results of the EVasCu Study and a Meta-Analysis Including EVasCu Data and Prior Studies
Source: Metabolites. 2025 Dec 24;16(1):20. doi: 10.3390/metabo16010020 (PMC12844247; doi:10.3390/metabo16010020)

Supplementary materials

Supplementary Table S1. Search strategy.

Adults **OR** Adult population **OR** Adult subjects	**AND**	Visceral adiposity index **OR** VAI **OR** New visceral adiposity index **OR** NVAI	**AND**	Arterial stiffness **OR** Pulse wave velocity **OR** PWV **OR** Aortic pulse wave velocity **OR** Brachial-ankle pulse wave velocity **OR** Carotid-femoral pulse wave velocity **OR** a-PWV **OR** ba-PWV **OR** cf-PWV

Supplementary Table S2. Quality assessment with the tool for observational cohort and cross-sectional studies of the National Heart, Lung and Blood Institute for the association between arterial stiffness and visceral adiposity index.

	Son et al, 2021	Ataee et al, 2023	Nakagomi et al, 2019	Li et al, 2021	Fan et al, 2021	EVasCu study
1. Was the research question or objective in this paper clearly stated?	Yes	Yes	Yes	Yes	Yes	Yes
2. Was the study population clearly specified and defined?	Yes	Yes	Yes	Yes	Yes	Yes
3. Was the participation rate of eligible persons at least 50%?	Yes	Yes	NR	Yes	No	Yes
4. Were all the subjects selected or recruited from the same or similar populations (including the same time period)? Were inclusion and exclusion criteria for being in the study prespecified and applied uniformly to all participants?	Yes	Yes	Yes	Yes	Yes	Yes
5. Was a sample size justification, power description, or variance and effect estimates provided?	No	NR	Yes	NR	No	NR
6. For the analyses in this paper, were the exposure(s) of interest measured prior to the outcome(s) being measured?	No	Yes	No	Yes	No	Yes
7. Was the timeframe sufficient so that one could reasonably expect to see an association between exposure and outcome if it existed?	No	NR	No	NA	No	NA
8. For exposures that can vary in amount or level, did the study examine different levels of the exposure as related to the outcome (e.g., categories of exposure, or exposure measured as continuous variable)?	No	Yes	No	Yes	No	Yes
9. Were the exposure measures (independent variables) clearly defined, valid, reliable, and implemented consistently across all study participants?	Yes	Yes	Yes	Yes	Yes	Yes
10. Was the exposure(s) assessed more than once over time?	No	No	No	NA	No	NA
11. Were the outcome measures (dependent variables) clearly defined, valid, reliable, and implemented consistently across all study participants?	Yes	Yes	Yes	Yes	Yes	Yes
12. Were the outcome assessors blinded to the exposure status of participants?	No	NR	No	NR	No	NR
13. Was loss to follow-up after baseline 20% or less?	Yes	Yes	NR	NA	No	NA
14. Were key potential confounding variables measured and adjusted statistically for their impact on the relationship between exposure(s) and outcome(s)?	Yes	Yes	Yes	Yes	Yes	Yes
Legend: NR (NO REPORTED); NA (NO APPLICABLE)						

Supplementary Table S3: Meta-regression models according to mean age, percentage of female, waist circumference and body mass index, HDL-cholesterol and triglycerides.

**Variable**	**Coefficient**	**95% CI**	***p* value**
**Age**	**0.01**	**0,007 - 0,013**	**0,001**
% female	-0,003	-0,015 - 0,009	0.551
WC	0.0004	-0,029 - 0,029	0.967
BMI	0.002	-0,111 - 0,115	0.962
HDL	-0.003	-0,049- 0,044	0.872
TG	-0.001	-0,007- 0,006	0.774

Supplementary Figure S1. Forest plot including the association between visceral adiposity index and central and peripheral arterial stiffness.

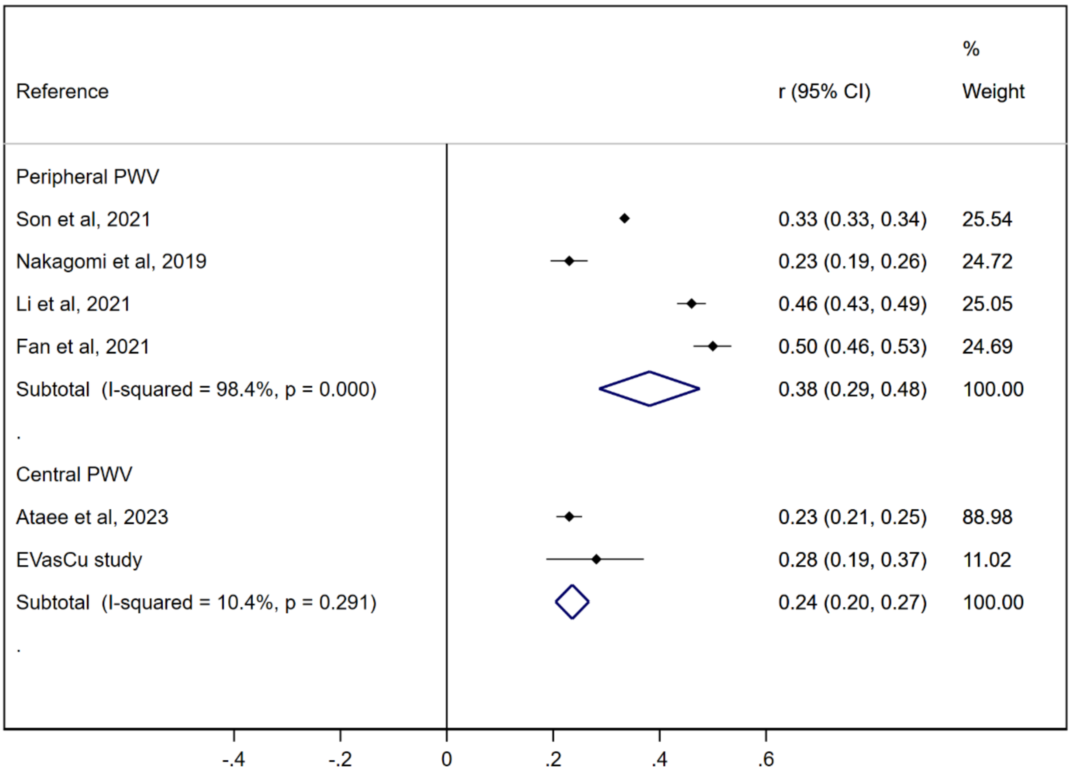

Supplement: Supplementary file 1 [file metabolites-16-00020-s001.zip › metabolites-4029621-supplementary.pdf]
